# Oral Bile Reinfusion in Chronic Percutaneous Transhepatic Cholangiodrainage

**DOI:** 10.14309/crj.0000000000000421

**Published:** 2020-07-10

**Authors:** Alyssa Kahl, Shruti Khurana, Scott Larson

**Affiliations:** 1 McGovern Medical School, The University of Texas Health Science Center at Houston (UTHealth), Houston, TX

## Abstract

Percutaneous transhepatic cholangiodrainage is an intervention for obstructive jaundice that, although effective in decreasing bilirubin levels, often leads to depletion of regular bile acids that subsequently cause malabsorption, diarrhea, and acute kidney injury. Bile reinfusion (BR) is a method of enteral refeeding of biliary secretions to replenish innate bile acids to the patient. In addition, BR is a low-cost alternative to exogenous bile acid replacement and abates the need for inpatient fluid resuscitation. We report oral BR in a patient with percutaneous transhepatic cholangiodrainage due to choledocholithiasis and review the literature on BR.

## INTRODUCTION

Choledocholithiasis is a common medical condition that develops in up to 20% of patients with gallstones.^1^ A small portion of stones within the bile duct can prove challenging to treat, with conventional methods such as endoscopic retrograde cholangiopancreatography (ERCP) because of both patient and stone factors.^2–4^ Owing to these difficulties, many endoscopists may opt for alternative treatments such as percutaneous transhepatic cholangiodrainage (PTCD). The total volume of bile secretions range from 500 to 600 mL per day.^5^ PTCD with higher outputs of over 2 L can lead to a deficiency of bile acids causing numerous problems such as fat malabsorption, diarrhea, acute kidney injury, and electrolyte abnormalities.^6^ Bile reinfusion (BR) replaces bile acids in the gut through oral refeeding of biliary drain secretions. Currently, evidence on the efficacy of BR is limited because there are only a few case studies and trials of BR in the setting of malignant obstructive jaundice.

## CASE REPORT

A 63-year-old white woman with Lynch syndrome, a history of ovarian cancer requiring hysterectomy and oophorectomy, gastric adenocarcinoma requiring subtotal gastrectomy and Roux-en-Y reconstructive surgery, previous cholecystectomy, and a history of left ventricular thrombus requiring anticoagulation with warfarin presented with 1 day of intermittent epigastric pain and emesis. Laboratory testing demonstrated direct hyperbilirubinemia of 3.3 mg/dL and elevated transaminases of aspartate aminotransferase of 463 U/L and alanine aminotransferase of 307 U/L. A right upper quadrant ultrasound showed a stone in the middle common bile duct (CBD) measuring 0.7 × 0.6 cm, causing mild biliary dilation of 0.9 cm.

After an appropriate reversal of anticoagulation, an ERCP was attempted with unsuccessful cannulation because of abnormal anatomy from previous surgeries. Percutaneous biliary drain placement by interventional radiology was considered a very high-risk procedure because of the patient's multiple comorbidities, including a chronic left ventricular thrombus requiring anticoagulation. A laparoscopic exploration was deemed technically difficult because of an extremely small gastric remnant left after the gastrectomy. Therefore, an open CBD exploration was performed by surgery for stone extraction and a 14 French percutaneous T tube was placed for temporary transhepatic cholangiodrainage.

On day 6 of the postoperative day, she developed dehydration because of large volume output from the T tube of up to 1.5 L per day with loose and greasy stools. This was accompanied with acute kidney injury with an increase in creatinine from 1.12 to 2.96 mg/dL. Owing to the concern for a biliary leak causing high biliary output, a T tube cholangiogram was unremarkable. BR was initiated by reconstituting the T tube output with juice in equal proportions, 4 times per day before meals. After 1 week on BR, the steatorrhea resolved. She showed a slow renal recovery, and her liver function tests normalized with a direct bilirubin of 0.4 mg/dL and transaminases of aspartate aminotransferase 32 U/L and alanine aminotransferase 23 U/L.

After 2 weeks, she came for a regular follow-up and had persistent large volume output from the T tube along with pain around the drain site. A cholangiogram revealed retained stones in the CBD and left hepatic duct with intermittent obstruction because of a stone immediately above the sphincter of Oddi. ERCP with a rendezvous procedure through the T tube was attempted, CBD was cannulated, and a 7-cm plastic biliary stent was placed. The patient continued BR for a total of 4 months until the stent placement. Four weeks later, a repeat ERCP was performed for stent exchange. The patient underwent a definitive stent removal 6 months later. At her most recent follow-up 2 months after her last procedure, the patient remained asymptomatic with a normal total bilirubin of 0.6 mg/dL.

## DISCUSSION

We describe a case of oral BR in a patient undergoing PTCD due to choledocholithiasis in the setting of altered gastrointestinal anatomy because of Roux-En-Y gastrojejunostomy and previous cholecystectomy. ERCP was pursued in her case, but her previous Roux-en-Y reconstruction proved difficult to cannulate, and a decision was made to pursue surgical intervention based on her risk of developing cholangitis. The large volume output of her T tube and worsening clinical status led to the decision to pursue oral BR. The patient was able to easily tolerate oral intake of bile well when mixed with juice or soda for palatability and continued to do so through the course of PTCD.

Bile is a complex solution that composes water, inorganic electrolytes, and organic solutes such as bile acids, phospholipids, cholesterol, and bile pigments.^5^ Bile acids serve many functions in the gastrointestinal tract such as facilitating digestion of dietary fats and fat-soluble vitamins and maintaining cholesterol homeostasis. They act as surfactants and emulsify lipids into micelles, which can then be broken down by lipases in the small intestine.^5^ Loss of bile acids from PTCD can impair fat digestion and thus causes steatorrhea. BR allows the reinstitution of these lost bile acids into the gut, thus avoiding malabsorptive diarrhea and its further complications.

BR has shown variable success as reported in a few case reports and small studies mainly focusing on patients with obstructive cholangiocarcinoma.^7–11^ Yu et al showed beneficial results through a retrospective study in patients with hilar cholangiocarcinoma receiving PTCD with BR vs conservative treatment alone before surgical resection.^7^ Song et al performed a similar study looking at patients undergoing PTCD with BR and whether they received enteral feeding, showing no improvement in postoperative recovery.^8^ Being retrospective in nature, both the studies may exhibit inherent selection bias for patients with poorer prognosis and did not compare between PTCD alone and PTCD with BR. Wang et al took it a step further to elucidate the possible benefits of BR by randomizing patients undergoing PTCD for malignant obstructive jaundice to receive BR or exclusive drainage and found that BR facilitated the recovery of hepatic function and serum protein levels.^9^

Although varied efficacy of BR has been noted in published studies, using the patient's own bile for treatment is more cost-effective than alternative therapies such as exogenous bile salts or intravenous (IV) fluid resuscitation. Tripathy et al demonstrated that both BR and exogenous bile salt administration hastened renal recovery in patients with obstructive jaundice but refeeding the patient's own bile comes at no additional cost.^12^ In addition to using our patient's own natural bile to their benefit, we were able to diminish further costs because our patient was able to continue BR at home and thus did not require continued hospitalization or readmissions for IV fluid resuscitation. The added costs of hospital admission, IV fluids, and any necessary bile salt replacement should be weighed against readily available, natural bile used in BR. BR demonstrates a more affordable and sustainable method of fluid repletion in patients with obstructive jaundice who would otherwise require added therapies and accrue further healthcare costs. Side effects including nausea, intolerance due to taste or volume of liquid intake, patient's support at home, their ability to follow instructions should be considered prior to BR.

To our knowledge, there are few case reports of BR in the setting of choledocholithiasis. In addition to the cases of malignant obstructive jaundice, other studies did use BR in the setting of biliary tract surgery-related injury and to increase cyclosporine absorption in patients who had received liver transplants.^13,14^ In conclusion, BR is a safe, low-cost intervention and should be considered in patients with obstructive jaundice who undergo PTCD and develop malabsorption, dehydration, worsening electrolyte derangements, or acute renal injury.

## DISCLOSURES

Author contributions: A. Kahl and S. Khurana wrote the manuscript and approved the final version. S. Larson edited the manuscript, approved the final version, and is the article guarantor.

Financial disclosure: None to report.

Previous presentation: This case was presented at the American College of Gastroenterology Annual Scientific Meeting; October 25-30, 2019; San Antonio, Texas.

Informed consent was obtained for this case report.
